# Qhali: A Humanoid Robot for Assisting in Mental Health Treatment

**DOI:** 10.3390/s24041321

**Published:** 2024-02-18

**Authors:** Gustavo Pérez-Zuñiga, Diego Arce, Sareli Gibaja, Marcelo Alvites, Consuelo Cano, Marlene Bustamante, Ingrid Horna, Renato Paredes, Francisco Cuellar

**Affiliations:** 1 Engineering Department, Pontificia Universidad Católica del Perú, San Miguel, Lima 15088, Peru; alvites.marcelo@pucp.edu.pe (M.A.); marlene.bustamante@pucp.edu.pe (M.B.); ingrid.hornav@pucp.edu.pe (I.H.); cuellar.ff@pucp.pe (F.C.); 2 Department of Psychology, Pontificia Universidad Catolica del Peru, San Miguel, Lima 15088, Peru; s.gibaja@pucp.edu.pe (S.G.); renato.paredes@pucp.edu.pe (R.P.); 3 Department of Art and Design, Pontificia Universidad Catolica del Peru, San Miguel, Lima 15088, Peru; consuelo.cano@pucp.edu.pe

**Keywords:** social assistive robot, mental health, humanoid robotics

## Abstract

In recent years, social assistive robots have gained significant acceptance in healthcare settings, particularly for tasks such as patient care and monitoring. This paper offers a comprehensive overview of the expressive humanoid robot, Qhali, with a focus on its industrial design, essential components, and validation in a controlled environment. The industrial design phase encompasses research, ideation, design, manufacturing, and implementation. Subsequently, the mechatronic system is detailed, covering sensing, actuation, control, energy, and software interface. Qhali’s capabilities include autonomous execution of routines for mental health promotion and psychological testing. The software platform enables therapist-directed interventions, allowing the robot to convey emotional gestures through joint and head movements and simulate various facial expressions for more engaging interactions. Finally, with the robot fully operational, an initial behavioral experiment was conducted to validate Qhali’s capability to deliver telepsychological interventions. The findings from this preliminary study indicate that participants reported enhancements in their emotional well-being, along with positive outcomes in their perception of the psychological intervention conducted with the humanoid robot.

## 1. Introduction

In recent decades, the applications of robots have become widespread across various domains, including manufacturing and industry, search and rescue, entertainment, education and research, assistance, and healthcare. Humanoid robots are characterized by their resemblance to the human body and often incorporate features such as a head, torso, arms, and legs. These attributes hold immense potential, particularly in the field of social robotics, where the aim is to develop machines capable of engaging in natural, socially intelligent interactions with humans, demonstrating behaviors that facilitate communication, collaboration, and companionship [1,2].

In this context, a social assistive robot (SAR) is used to assist by creating a close and effective interaction with a human user [3,4]. During the context of the COVID-19 pandemic, social interactions related to commerce, education, and health services had to be redesigned to maintain functionality without risking human health. In most cases, social robots were introduced as reliable alternatives to “the new normal” and its novel demands [5,6,7].

In [4], the authors evaluated how a robot’s appearance influences patients’ acceptance levels of it as well as related medical procedures. Physical features are crucial in the design of social robots; this entails how people would perceive the effectiveness of the device, as studied in [8]. Indeed, anthropomorphic animal social robots, as suggested in [9], could present an appealing alternative to humanoid robots in the role of future companions for hospital treatment. However, these could be considered toys for child patients to avoid being viewed as less effective than other options by adults. Moreover, the robot design process must consider features of hospital or care environments to guarantee prolonged usability [4]. Therefore, functionality could not be fully scrolled by the robot’s appearance.

Incorporating anthropomorphism in robots demonstrates positive outcomes in mitigating anxiety, stress, and negative perceptions linked to human–robot interaction (HRI) [10,11]. Human communication significantly relies on non-verbal components that vary across cultures and ages. Standardizing these gestures for social robots, as proposed by [12], is crucial. The user’s experience with social robots is shaped by humanoid shapes and movements as well as the presence or absence of facial expressions, impacting perceptions of trustworthiness. Striking a balance between facial anthropomorphic similarities and perceived naturality is essential, given humans’ tendency to attribute lifelike properties to machines. Subtle facial expressions, as discussed in [13,14], influence how people perceive the reliability and motivations of robots. Furthermore, the assumed gender of humanoid robots, as studied by [15], plays a role in shaping attitudes during interactions.

Additionally, it enhances feelings of familiarity toward the robot [16]. Moreover, research indicates that robotic corporal expressions contribute to a better understanding of the robot’s emotional state [17]. Similarly, to generate appropriate facial expressions, recognizing the user’s facial expressions is worthwhile. In Jubileo’s case: It is an open-source, 3D-printed robot with an animatronic face capable of reproducing the user’s facial expressions, both in reality and within its own virtual simulation environment. By doing so, the interactive user experience is enriched in terms of reliability and immersion [18].

One complementary feature of HRI is the ability of social robots to speak as well as understand the intention of the message, in order to provide more consistent feedback to their human interlocutors. Along with Jubileo’s case, in [19], there was an initiative to work on a natural language understanding (NLU) task through deep learning techniques, but applied in a commercial robot.

In the field of mental health, SARs (socially assistive robots) can serve a multitude of roles, ranging from diagnosis to treatment and rehabilitation [20]. In the diagnostic domain, social assistive robots have demonstrated efficacy in assessing mental, psychiatric, and neurological disorders, as indicated by research such as that conducted by Rasouli [20]. The ability of these robots to engage with users on a social and emotional level enhances the diagnostic process, offering an understanding of an individual’s mental health.

Recent research underscores the positive impact of SAR on treatment outcomes [21]. These robots have been effective at improving mood, reducing anxiety symptoms [22], and enhancing overall well-being and perceived social support [23,24]. This suggests a promising pathway for incorporating these robots into therapeutic interventions, aiming to increase the choices available for both receiving and delivering mental healthcare.

Beyond treatment outcomes, the role of social assistive robots in rehabilitation, both physical and cognitive, is notable [25]. These robots showcase the versatility in aiding rehabilitation efforts for both children and adults. The interactive and supportive nature of the robots contributes to more engaging and effective rehabilitation [26].

Commercial robots (like Nao, Pepper, and Buddy) have demonstrated success in accompanying and supporting both children and older adults, leading to positive outcomes, as indicated by [24]. These findings suggest that social assistive robots can play a crucial role in providing companionship and emotional support in mental health contexts. However, despite all the research conducted, it is essential to acknowledge challenges and areas that need further exploration. These may include refining the integration of robots into existing mental healthcare frameworks, addressing ethical considerations, and exploring long-term effects on user engagement and outcomes.

Nevertheless, even if it seems simpler to just use a commercial social robot instead of designing new models, this design process is worthwhile because it allows us to add specific features aimed at medical purposes or patient requirements.

One challenge in designing humanoid robots for interaction with humans is the necessity for expressive bodily movements. These movements aim to evoke the expected emotional responses from individuals during interactive scenarios. Therefore, body language plays a crucial role in human communication, constituting over 50% of conveyed information [27]. Expressive bodily movements can independently communicate information and significantly impact human emotions. In the context of social robots, especially those lacking facial features, designing emotional body language poses a challenge [17,28]. Research indicates that robot movements influence attitudes and perceptions, enhancing understanding of robot emotions, trustworthiness, and eliciting empathetic responses. Combining robotic speech and movements promotes familiarity and fosters human-like interactions, emphasizing the importance of robotic movement in both functional and social aspects of interaction [29]. Another important source of non-verbal communication is the robot’s posture. Research highlights that posture can transfer symbolic messages of the user’s attitude or intention during interaction [30,31]. Furthermore, changes in body posture could reflect people’s affective states [32]. For example, when the speaker is slouched with their arms folded, it usually conveys a degree of relaxation [32], while a rigid torso in one direction is generally interpreted as indicating the locus of attention [33].

Some researchers have established axioms and their consequent principles along their conceptual designing process of a SAR. Even when the services offered by robots might vary, SARs should be influential in the user’s intrinsic motivations and customizable enough to keep the user’s engagement and trust levels. Therefore, in [34], five general design principles for any SAR-based therapeutic intervention were instituted: being motivating, fluid and highly interactive, personable, intelligent, and task-driven. Successful acceptance experiences with a proposed SAR exercise system for elderly users, who were the target audience in that study, supported those applied approaches.

At Pontificia Universidad Católica del Perú (PUCP), the Qhali robot was designed, implemented, and validated as a socially assistive humanoid robot. Qhali, created for telepsychological interventions, features a humanoid design with two articulated 4DOF arms and a 2DOF head, including two (02) LCD screens. These components enable the robot to express gestures through the arm and head movements, enhancing the human–robot interaction during interventions. The robot is equipped with a navigation system for autonomous movement and audio-video systems to verbally and non-verbally communicate, express emotions, gestures, and body language, and enhance overall interaction. First, the 3D modeling and mechatronic design processes are developed, along with an approach to the non-linear control strategy to be implemented for the degrees of freedom (DOF) in the robotic arms [35]. Then, the psychological intervention proposal and its corresponding conceptual and technical requirements are defined. This included remote services features for doctor–patient interactions through video conferences and teleoperations of Qhali robot movements. Subsequently, a preliminary validation of Qhali robot gesture expression features, using CAD software-generated videos of Qhali robot non-verbal performances, was developed. The sample was composed of STEM-related participants who were asked to identify the gestures and their communicative meanings shown in the videos. Additionally, in [28], Godspeed metrics for evaluating participants’ subjective impressions about the robot were applied, where likability and perceived intelligence features registered the highest scores.

In this paper, we provide the following main contributions:

The first contribution is a comprehensive overview of the entire design process of the humanoid robot for assisting in mental health treatments. We initially outline the industrial design stage based on five (05) phases, ranging from initial research to implementation. Subsequently, we present the design and implementation of all hardware and software components, including the control platform. The second main contribution is related to the validation of the Qhali robot through a pre-experimental study, representing the first validation experience of the capabilities of this humanoid robot in performing psychological interventions.

The paper is organized as follows: Section 2 presents the description of the complete design process of the humanoid robot. Section 3 describes the hardware and software components. Section 4 details the experimental tests of the robot in operation. Section 5 analyzes the results found in the pre-experimental study. Finally, Section 6 concludes the paper.

## 2. Description of the Industrial Design Process for the Humanoid Robot

The industrial design process of a humanoid hospital robot was developed in five key phases, each significantly contributing to the final product [36].

### 2.1. Research Phase

**Figure 1:**
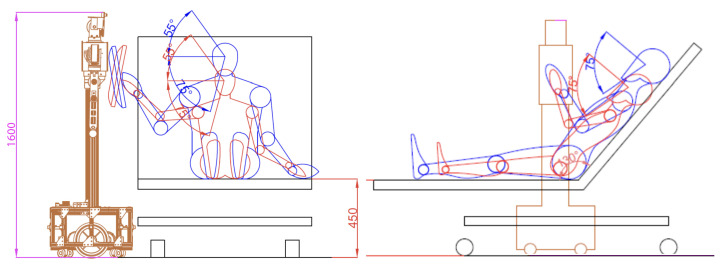
Ergonomic analysis of a patient in a seated position, focusing on extreme percentile ranges; front and side views.

The first step involved in-depth research to understand and empathize with the emotional and functional requirements of patients. This stage was crucial to ensure that the robot’s design achieved technical requirements and established an emotional connection with patients. The goal was to make the robot appear reliable and friendly, providing assistance and empathy, and establishing trust. Functionally, the robot was required to facilitate effective communication between the patient and the health specialist, even at a distance, through the transmission of sound and images. It was essential for the robot to adapt to various physical conditions of patients, whether standing, sitting, lying down, or with limited mobility, as shown in Figure 1.

### 2.2. Ideation Phase

**Figure 2:**
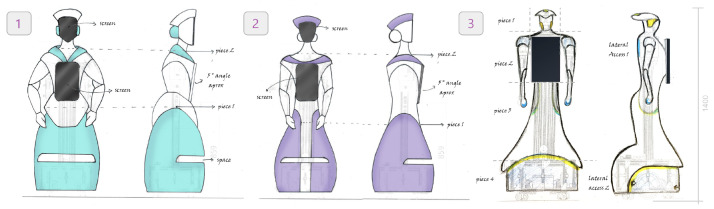
The design focus focused on creating natural, human-like forms and organic shapes, as well as a closeness to a hospital environment.

In this stage, creative ideas for the robot’s design were generated, based on the findings of the research. The focus was on conceptualizing a robot that could seamlessly integrate into the hospital and human environment. A design was envisioned that conveyed kindness and warmth, similar to the appearance of a nurse, with organic shapes and curves. This phase was essential to define the aesthetic and emotional directions of the project, creating a clear vision of the look and feel the robot should convey [37] (see Figure 2).

### 2.3. Design Phase

**Figure 3:**
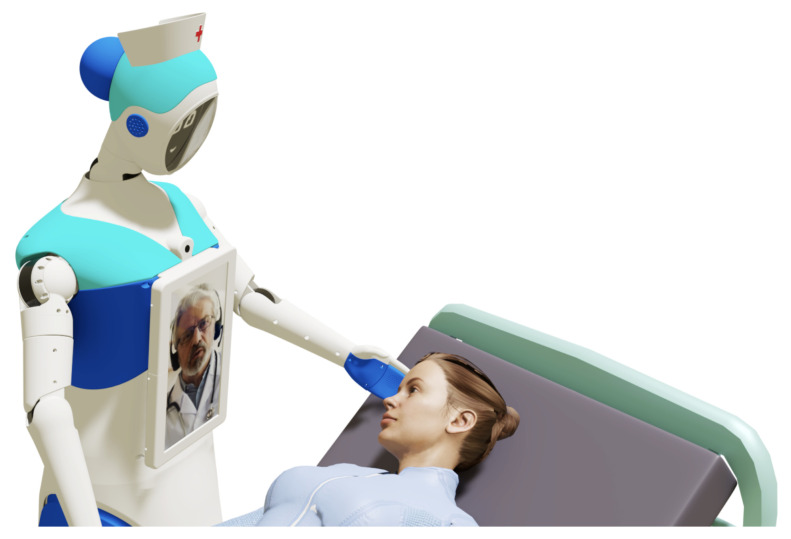
Arrangement of designated parts according to the ergonomic analysis.

In this phase, the concepts developed during the ideation process were actualized. A detailed physical design of the robot was developed, incorporating features such as a screen to display facial expressions and articulated arms for precise movements. An ergonomic study was also conducted to determine the best location for the screen, microphones, and speakers, ensuring comfortable and effective patient interaction (see Figure 3). Special attention was paid to functionality, ensuring that the final design was practical and safe, as well as met all the previously identified requirements [38].

### 2.4. Manufacturing Phase

**Figure 4:**
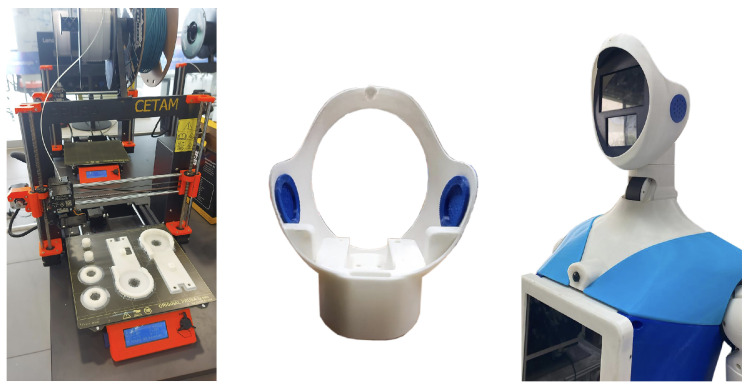
The manufacturing process of the Qhali robot.

The fabrication of the prototype of the robot involved using 3D printing technology to create the components of the casing, employing PLA material, and using Creality (Shenzhen, China) and Prusa (Prague, Czech Republic) printers. The parts were manufactured in sections according to their shapes and then carefully assembled. This phase was fundamental in bringing to life the design conceptualized earlier, transforming ideas and blueprints into a tangible product, as shown in Figure 4.

### 2.5. Implementation Phase

The final stage of the process was the implementation, which involved integrating the casing with the robot’s electronic components and structural frame. During this phase, significant adjustments were made to optimize the fit and alignment of the parts, ensuring the robot’s functionality and comfort for patients. This step was crucial to ensure that the robot not only functioned as intended but also met all quality and safety expectations.

In summary, each phase of the hospital robot’s industrial design process was meticulously planned and executed, contributing to a final product that not only satisfied technical and functional requirements but also established an emotional connection with patients, enhancing their experience in the hospital environment. This comprehensive approach ensured a design that was both innovative and empathetic, a clear reflection of the end-users requirements and expectations.

## 3. Design of the Hardware and Software Components Used in the Robot

### 3.1. Hardware Design

**Figure 5:**
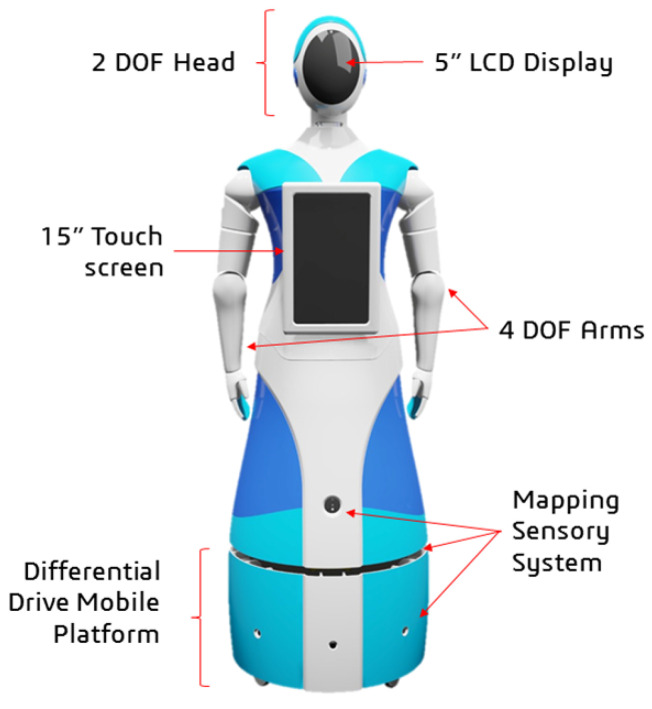
A 3D model of a humanoid robot and composition.

Qhali was designed with a deliberate blend of human-like and machine-like characteristics, aiming to embody an organic appearance reminiscent of the human form while integrating advanced technological functionalities. Standing at a height of 160 cm, the robot mirrors the average human stature, reflecting meticulous attention to detail in its construction. The internal structure encompasses a mobile platform with a differential drive system, complemented by a mapping sensor setup, and a humanoid mechatronic structure composed of two articulated arms, one touch screen on the chest, and an articulated head with a display (see Figure 5).

**Figure 6:**
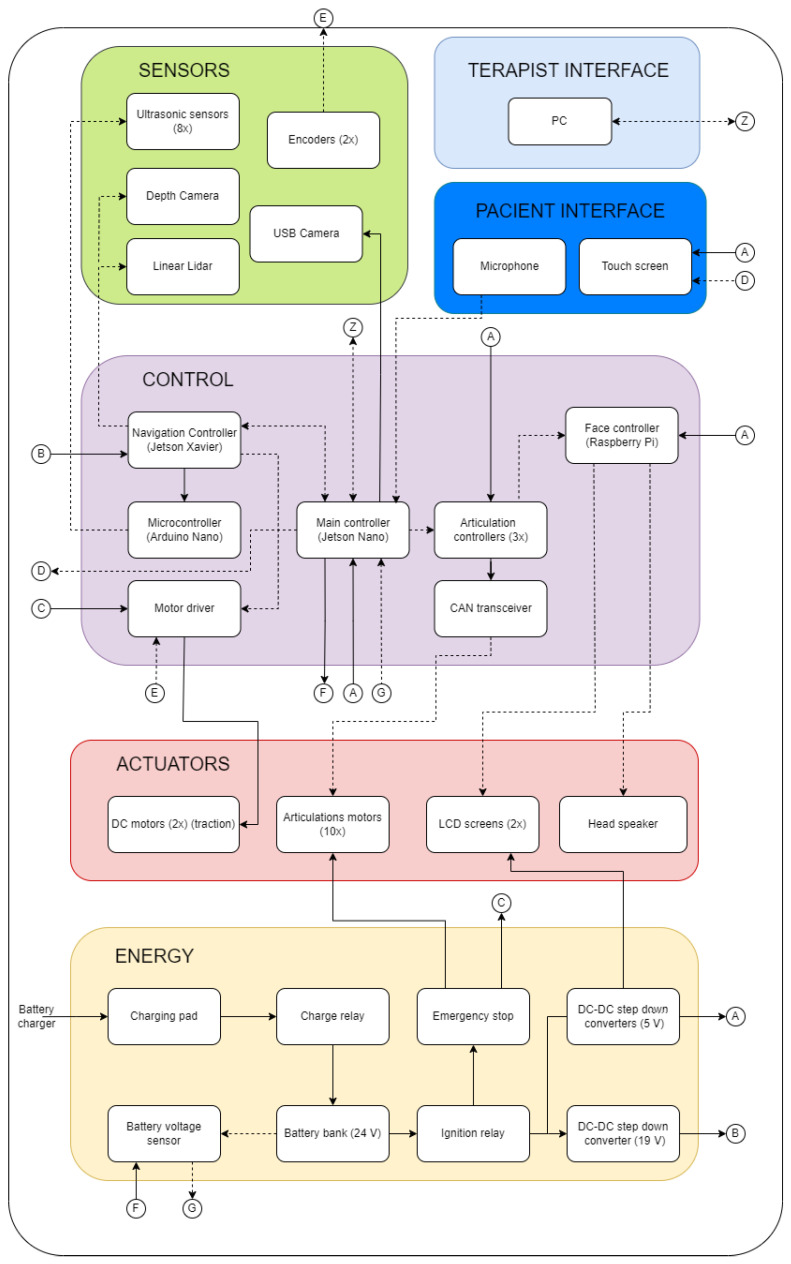
Hardware architecture of the robot.

The mechatronic system of the robot is composed of sensors, actuators, control, energy, and interface elements. Figure 6 shows the hardware architecture with the links of all the main internal components.

### 3.2. Mechatronic Articulations Design

**Figure 7:**
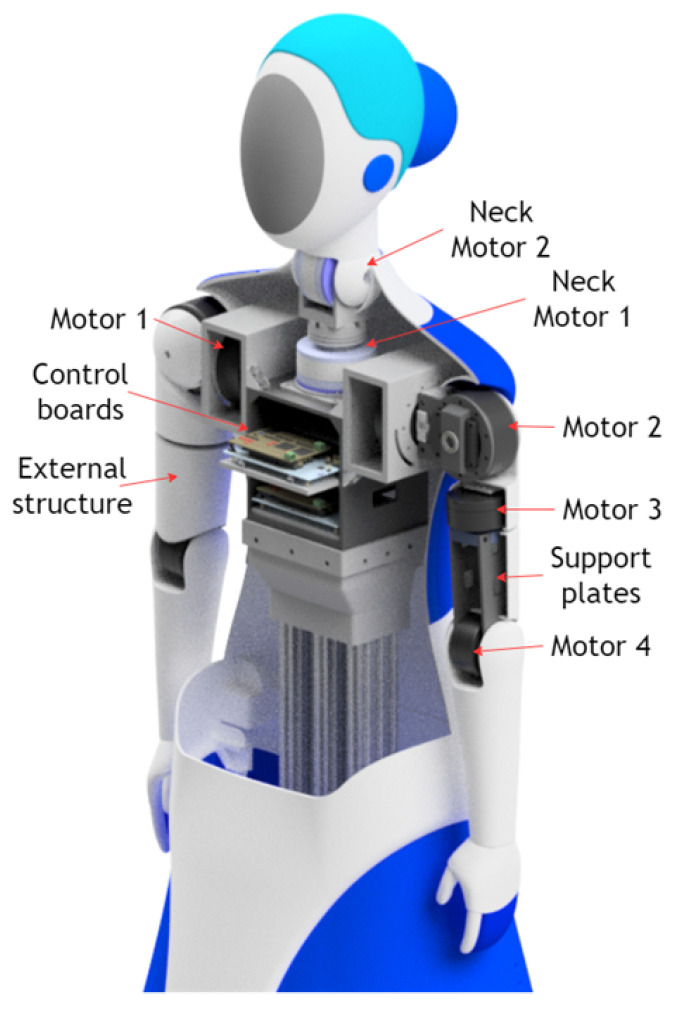
Internal design of articulate arms and head.

The robot’s articulations include two arms with four degrees of freedom (4-DOF), each driven by precise servomotors that enable nuanced movements (see Figure 7). These arms offer a wide range of motion: two (02) shoulder joints for abduction and flexion/extension, one (01) mid-arm joint for rotation, and one (01) elbow joint for forearm articulation. Carefully designed 3D-printed PLA structural components reinforce load-bearing capabilities while keeping the arm weight at 2 kg each. Additionally, the primary chest piece functions as foundational support for the head and initial arm joints, facilitating seamless integration with the robot’s upper body.

The head of the robot incorporates a 2-degree-of-freedom (2-DOF) articulation system powered by two (02) servomotors. Positioned strategically, one servomotor enables horizontal movement beneath the neck for rotational flexibility, while the other, situated vertically on the neck, allows the head to flex and extend. Complemented by a speaker for the voice, the head houses 5-inch and 3.5-inch LCD screens that enhance its communicative abilities. Primarily constructed using 3D printing techniques, the head’s structure features a durable thermoformed acrylic screen cover, ensuring longevity and functionality.

This amalgamation of components empowers the robot to express diverse gestures, combining arm and head movements with visual displays on the head and chest interfaces, significantly enhancing its communicative capabilities in interactive environments.

### 3.3. Differential Drive System Design

**Figure 8:**
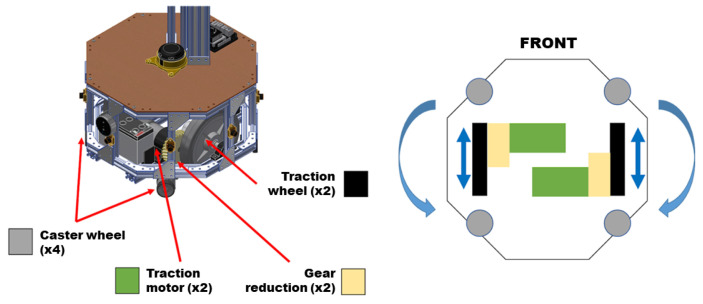
Differential drive system composition.

The robot is equipped with an automated movement system driven by wheels. Its traction system employs a differential distribution mechanism (see Figure 8).

This differential setup enables two distinct types of movement: linear motion, allowing forward or backward movement, and rotational motion around its axis in either a clockwise or counterclockwise direction. The traction system functions seamlessly, comprising four key components: support pulleys, traction wheels, a gear system, and traction motors. Four (04) caster wheels bear the weight of the robot while facilitating multi-directional movement. Two (02) traction wheels transmit movement generated by the pair of traction motors. Additionally, two (02) gear systems positioned between the motors and drive wheels act as reduction boxes, enhancing torque by reducing the motor rotation speed, thereby enabling efficient movement of the robot.

### 3.4. Sensing System Design

**Figure 9:**
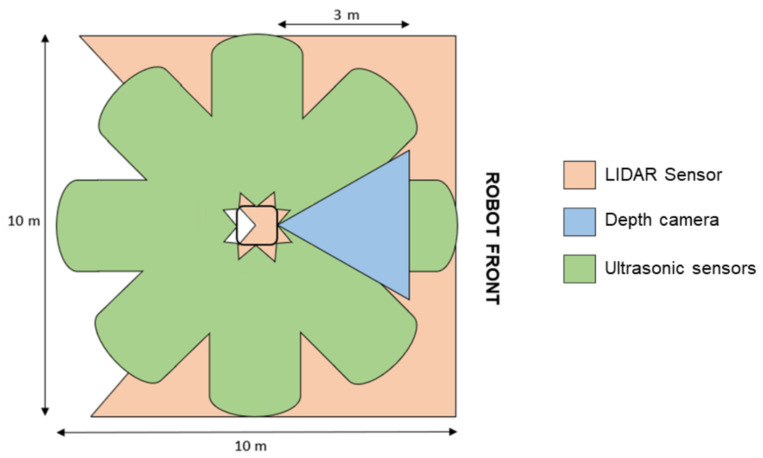
Top view of the robot that includes the field of vision of the components of the sensor system.

The robot’s navigation system, which allows it to move in environments with limited space and frequent moving obstacles, is composed of a sensing system of 360° cameras and sensors located in the upper front part to locate the patient and in the lower part to move through dynamic environments (see Figure 9). The sensing system is composed of one (01) depth camera, one (01) LIDAR sensor, and an array of eight (08) ultrasonic sensors.

The depth camera is located at the front of the robot to visually detect and identify obstacles in front of the robot and the areas through which the robot can move. This camera has an operating range of 0.25 to 9 m, a depth resolution of 1280 × 720, 30 FPS, and a depth field of 70 × 55. The Lidar sensor, located at the base of the robot (at a height of 40 cm above ground level), is used to identify static and dynamic obstacles that are in the path of movement and on the sides of the robot. This sensor has a working range of 0–360°, a scanning range of 0.2–25 m, and a scanning frequency of 5–15 Hz. The array of ultrasonic sensors is used to detect obstacles in the robot’s environment, on the front, sides, and rear area, with a measurement range of up to 10 m.

A strategy involving the use of multiple sensors utilizing different technologies for environmental identification was carried out in order to have adequate identification of static and dynamic obstacles [39]. Sensors, by using different technologies, provide different types of information (regarding the resolution, frequency, and amount of data) that complement each other to obtain an adequate mapping of the environment.

### 3.5. Control Systems

Since the robot consists of several elements, four (04) types of controllers are needed to command and receive information from all the elements. The main controller (Jetson Nano, Nvidia Corporation, Santa Clara, CA, USA) is used as a master controller to execute the different routines and actions of the robot, based on the information received through the interface. A dedicated navigation controller (Jetson Xavier, Nvidia Corporation) is used to execute the navigation algorithms, command the movement of the motors for the robot’s movement, and process the information from the sensing system. Three (03) articulation controllers (STM32, STMicroelectronics, Geneva, Switzerland) are used to command the articulated arms and head, one for each. These controllers execute pre-programmed routines based on setpoints received by the master controller. Finally, an additional controller (Raspberry Pi 4, Raspberry Pi Foundation, Cambridge, UK) is used to execute the facial expressions (see Figure 6).

The navigation controller is based on a two-level architecture: a local planner that aims to locally reach the positions established by the global planner. For this, the dynamics present in the navigation environment are taken into account, including fixed and moving obstacles measured by fusing the different sensors of the system. The local planner has been developed using algorithms rooted in reinforcement learning, allowing for changing environments to be managed through the analysis of multiple scenarios (actions and observations), penalties, and rewards, imitating how humans can perform the task, imitating the same process of learning based on the collection of experiences.

The articulation controllers also feature a two-level architecture; at the lower level, the control strategy used is based on the information provided by an encoder detailing the position and speed of each motor, along with internal torque regulation via a PI controller, which receives setpoint signals from a non-linear controller based on the backstepping strategy for trajectory tracking; this strategy also uses a velocity profile for arm and head movements [35,40].

### 3.6. Ergonomic Movements Design

**Figure 10:**
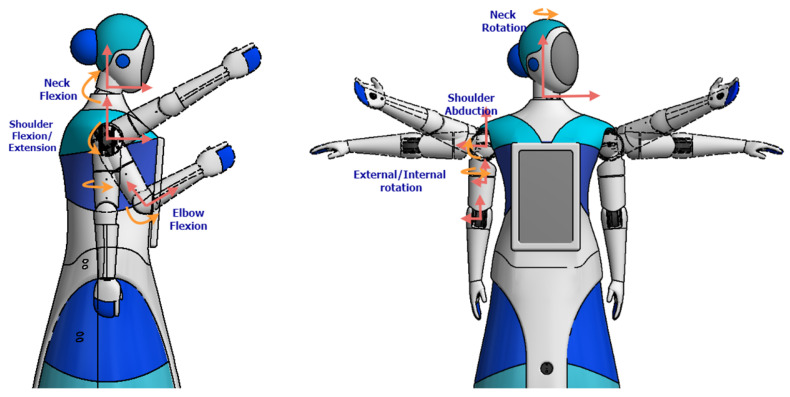
Ergonomic movements with articulate elements.

The Qhali humanoid robot features cognitive and ergonomic movements that help it express itself during its interactions with the patient. For this reason, it has two arms and an articulated head with two (02) screens to recreate facial expressions. A non-linear control scheme for trajectory tracking and a speed profile that executes the arm and head movements of the Qhali robot with natural motion was presented in [35]. Figure 10 shows the movements that the robot can perform with the arm and head joints it is equipped with. The arms have 4-DOF and the head 2-DOF, so they resemble the movements of a person.

**Figure 11:**
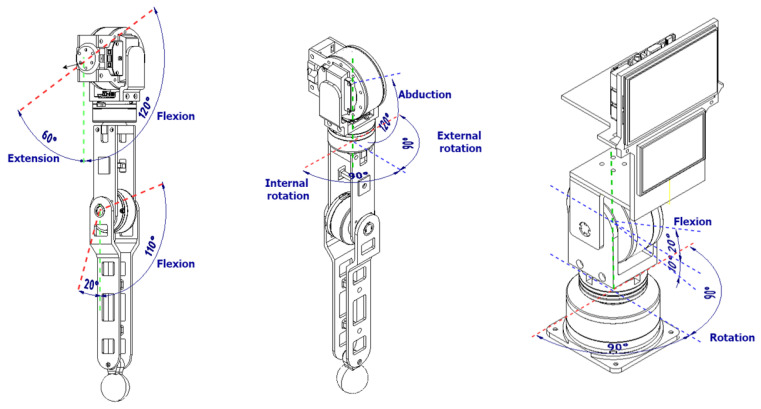
Detailed movement ranges executed by the robot.

Likewise, Figure 11 and Table 1 detail the capability of the movements that the robot can perform by making and using mobile articulation. The robot can execute six types of movements using the head, neck, shoulder, forearm, and elbow. The movement ranges are detailed for each type of movement that the robot can perform.

**Figure 12:**
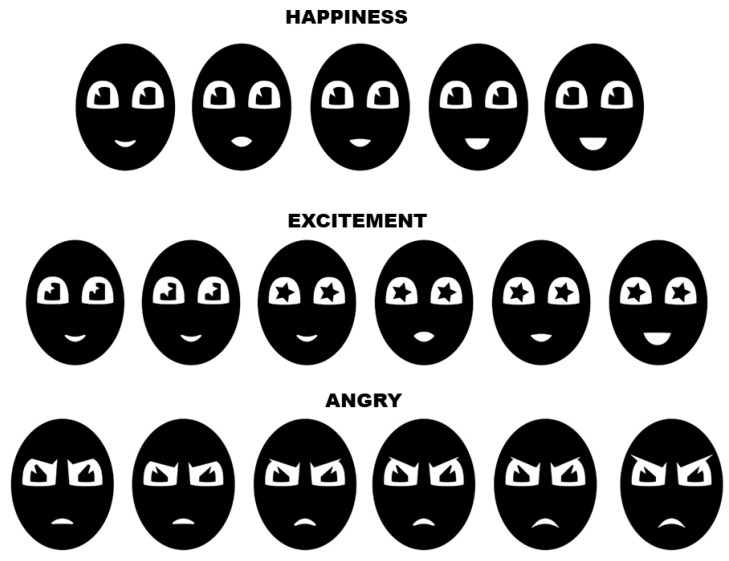
Facial expressions on the robot’s face.

Furthermore, by using the screens located on the robot’s head, it is possible to replicate facial expressions to complement the movements performed by the robot and express a certain feeling. Figure 12 presents some of the facial expressions designed for the robot to reproduce through the screen.

### 3.7. HRI System Design

**Figure 13:**
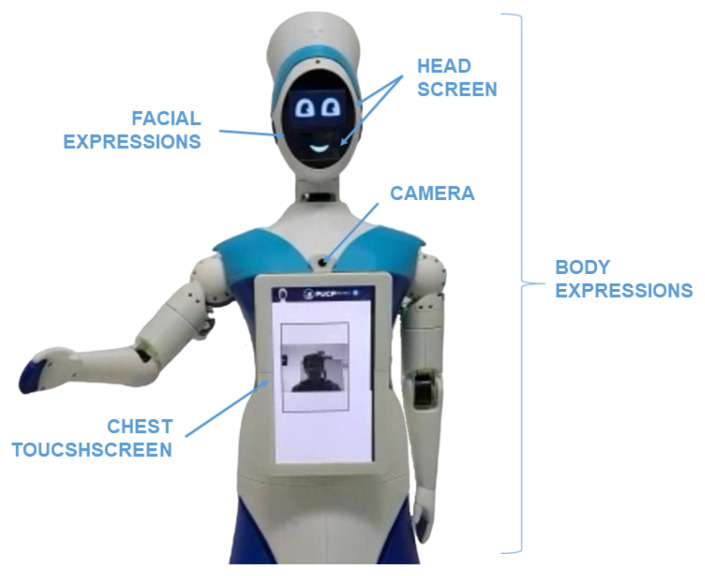
Elements for human–robot interaction.

The robot has two (02) screens in the head section and one (01) screen in the chest section to perform telepresence communication (as shown in Figure 13). To begin the psychological treatment, the robot must be positioned in front of the patient, who might be lying on a hospital stretcher in a room with several patients; therefore, it is necessary to guarantee privacy between the therapist and the patient through an adequate approach. This key functionality for distance communication that the Qhali robot can offer is achieved through the various movements that it can perform with its body, arms, and head. In addition, there is a touch screen on the chest for interacting with the patient, through which, tests, surveys, or other data requested by the therapist can be filled out. It also serves to display the video image of the therapist during the psychological intervention, who can also observe the patient using a camera located above the chest screen.

The robot also includes two (02) speakers on its head in order to express sounds when performing interaction routines and communicate with the patient when using distance communication. A test was performed using a sound level meter, measuring the decibels emitted by the robot at a distance of 1 m, in order to validate the sound level emitted by the robot. This test was performed in an empty environment (as it will be used for private sessions) and in a crowded environment (as it will be used for promotion routines). The results of the measurements indicated that the robot emitted sound ranging from 42.4 to 48.7 dB in the first environment and from 46.7 to 53.9 dB in the second environment. Additionally, for private sessions in crowded environments, it is recommended to use wireless Bluetooth headphones, which are compatible with the robot controller.

### 3.8. Web Interface Design

**Figure 14:**
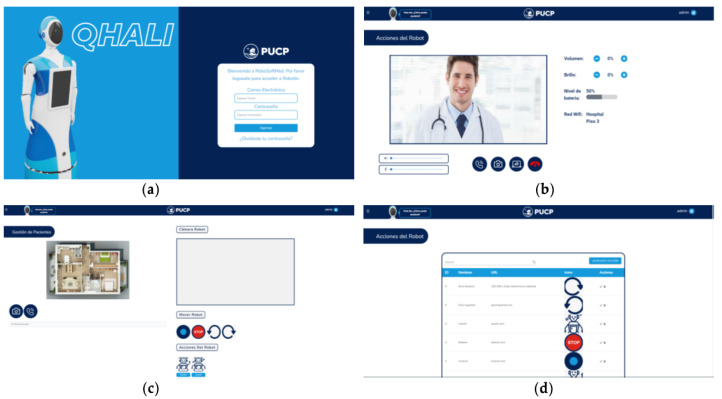
Teleoperation interface main screens: (**a**) access; (**b**) video conference and robot control; (**c**) remote teleoperation; and (**d**) configuration of robot actions.

For the proper teleoperation of the robot, it is necessary to design a platform with robust communication that ensures smooth transmission without latency, delays, or failures that may impact the system’s performance [41,42]. The web platform allows health specialists to control all the functionalities of the robot, including the execution of the therapeutic session. Figure 14 presents the design of the web platform.

### 3.9. Security System

The programming of the robot’s master controller has been designed to stop movement at any time a malfunction is detected. For instance, the robot stops in the following cases:

- If the arms or head become stuck or impact any object, a peak in current is measured and the movement is disabled.
- If during the robot’s movement, it encounters an object on the ground, a variation in current is detected and the motors are stopped.
- If the robot is moving and the sensing systems detect a nearby object that could impact the robot, the motors are stopped.

Additionally, at any time, the operator can stop the robot through a web interface via a virtual stop button, as the robot is teleoperated and monitored for its use. In addition, the robot includes a physical stop button on its back in emergencies. The physical button could be used by a nurse or an assistant who must be present during the robot’s operation.

Moreover, from the perspective of view of hardware fault diagnosis, fault detection and isolation algorithms are implemented in critical system components, such as actuators and sensors, through the use of model-based fault diagnosis methods to calculate analytical redundancy relations (RRAs) by applying distributed and decentralized architectures (see details in [43,44]).

From the perspective of safeguarding patient privacy, a video conferencing system has been implemented to exert complete control over the information transmitted through the telecommunications infrastructure. Additionally, the generated database was used in compliance with privacy and data policies, in accordance with the current regulations of restricted use for the research project.

**Figure 15:**
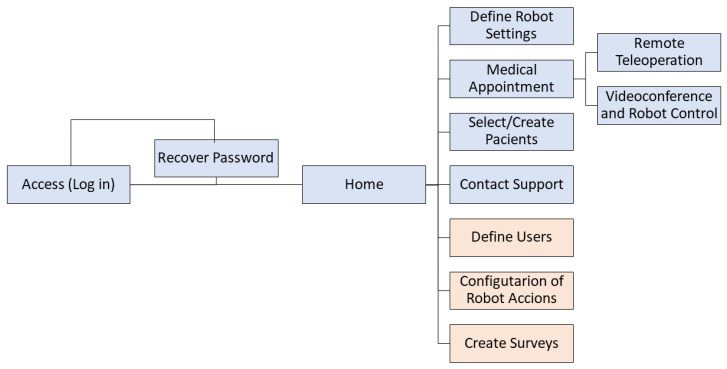
Screen flow diagram of the web interface.

A diagram representing the flow between the windows is shown in Figure 15. The colors represent the types of users. The light blue color represents both the therapist and the administrator, while the orange color only represents the administrator. After completing the actions in the modules, one can return to the home window through the Qhali icon in the top navigation bar.

For example, there is a database of patients that contains their basic information, as well as their registered medical histories. Also, the robot and patient can be selected to initiate the intervention; the specialist then starts the video call, where a conversation is established with the person. In addition, for robot control, two sets of buttons are shown, actions and movements. Actions refer to a composition of predefined movements involving the arms and head, changes in facial expressions, and recorded dialogues so that the robot can express what the therapist prefers. On the other hand, movements refer to the translation or rotation of the whole robot, with a differential drive mechanism. Moreover, there is a section on the platform where customized surveys are registered; in order to answer, the patient uses the Qhali touch screen. These surveys are added to the respective person’s medical history.

## 4. Testing the Qhali Robot in a Controlled Environment

A behavioral pre-experiment was conducted to validate Qhali’s capability of conducting a telepsychological intervention. We recruited *N* = 13 university students. The majority of participants (61.53%) were women. Participant ages ranged between 18 and 27 years (*M* = 22.54, *SD* = 3.55). The majority were aged between 18 and 23 years (84.61%), and the remaining participants were aged between 24 and 27 (15.38%). People with neurological disorders or visual/auditory disabilities were excluded from the study. Participants were asked to complete a questionnaire encompassing basic personal data. Due to the nature of the study, at least one emergency contact was also requested to address potential emotional crises during the intervention.

**Figure 16:**
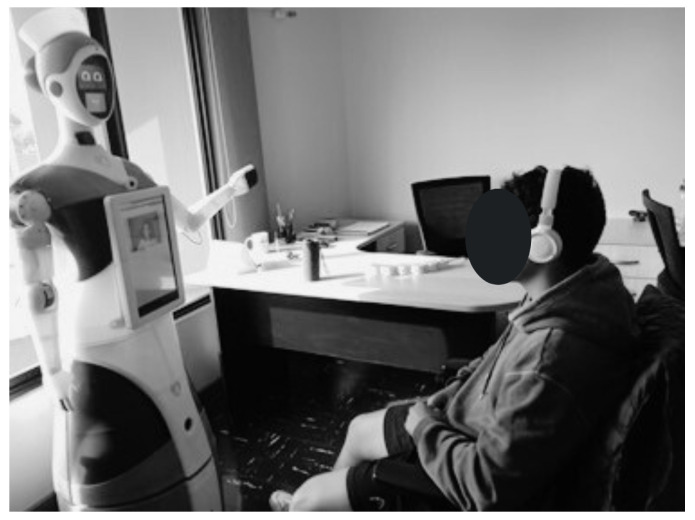
Qhali performing her routine to a research group member.

After providing all the necessary information, participants were escorted to a secluded room where the robot was located. Participants were asked to sit in front of the robot (as shown in Figure 16). Immediately after, Qhali began a routine that consisted of gestures and phrases crafted from HRI literature [31,33,45,46], with collaboration from an experienced entertainer professional. The routine started with Qhali’s introduction and transitioned to the introduction of the therapist. Following this, the robot powered down, and the therapist initiated contact with the participant, marking the beginning of the psychological intervention. This consisted of a brief recognition of difficulties that the participant may have encountered in the past week. During this session, situations that could have elicited emotional discomfort were not addressed. Moreover, it is important to mention that an emotional containment protocol was developed to address instances where participants displayed signs of psychological distress during the session. This protocol entailed offering guidance rooted in dialectical behavioral therapy (DBT) to alleviate the symptoms. In cases of severe distress, the therapist would provide psychological support to help manage the participant’s emotional state. Additionally, the therapist would advise participants to consider engaging in a therapeutic process. Upon completion of the intervention, the Qhali robot reactivated, displaying a goodbye message to the participant.

Once the interaction was over, participants were escorted to another room to complete a new questionnaire. First, they were presented with the question: ‘In case you receive psychological attention, would you prefer it to be through Qhali robot?’. Next, to collect their subjective impressions about the Qhali robot, participants were asked to complete one of the most frequently used questionnaires in the field of HRI: The Godspeed questionnaire [47]. This scale, comprising twenty-four (24) items, captures five (05) dimensions: anthropomorphism (ANT), animacy (ANI), likeability (LIK), perceived intelligence (INT), and perceived safety (SFT). In addition, participants completed a four (04)-item questionnaire assessing the communicative attributes of the robot on a 7-point Likert scale [48]. This scale explored the speed (SPD), attractiveness (ATT), ease of understanding (UND), and expressiveness of the robot (EXP).

To evaluate satisfaction with the treatment received, the Consumer Reports Effectiveness Scale (CRES-4) was used [49]. Participants responded to a four (04)-item questionnaire to capture three main components: satisfaction, problem-solving, and perception of emotional change. Finally, participants were posed with the question: “How comfortable would you be with the use of robots to provide psychological care?”. On a 5-point Likert scale, participants could select from “Not at all” to “Completely agree”.

Data analysis was performed using the Python programming language. The Pingouin v.0.5.1 [50] and Seaborn v.0.11.2 [51] packages were utilized for statistical analyses and visualizations, respectively.

## 5. Results

At a descriptive level, it was observed that a significant majority of participants (84.64%) expressed a preference for receiving a psychological intervention through the Qhali robot. Additionally, a substantial percentage (76.92%) fully agreed that utilizing robots for delivering psychological interventions is a viable option.

**Figure 17:**
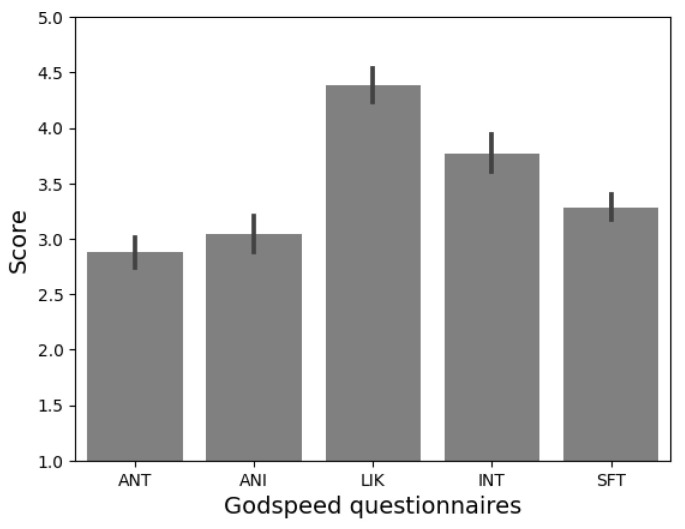
Responses to the Godspeed questionnaire. Participants found the robot’s likeability and perceived intelligence to be slightly prominent.

Prior to this study, a preliminary experiment was conducted to evaluate users’ perceptions of Qhali’s attributes and gestures [28]. Participants were presented with an animated video of Qhali to elicit their responses in the Godspeed Questionnaire and communication attributes scale (see Figure 17). Consistent with the findings from the mentioned study [28], participants highlighted the robot’s likeability (*M* = 4.38, *SD* = 0.53), perceived intelligence (*M* = 3.76, *SD* = 0.6), and perceived safety (*M* = 3.28, *SD* = 0.4) as particularly noteworthy, distinguishing them from the other two dimensions of the questionnaire: U (*M* = 3.08, *SD* = 0.58) and anthropomorphism (*M* = 2.87, *SD* = 0.4).

The elevated scores in the likeability dimension can be attributed to the robot’s design. The robot had a balanced appearance between human-like and machine-like, as revealed by the anthropomorphism scores. This presumably allowed for affective proximity with users [52]. Moreover, the speech displayed by Qhali during the routine was not only informative but also infused with playfulness, employing rhetorical and entertaining elements. These characteristics might have contributed to enhancing Qhali’s user-friendliness [53]. Additionally, the elevated scores in the perceived intelligence of the robot could be attributed to the perceived complexity and realism of the executed gestures and speech [54].

**Figure 18:**
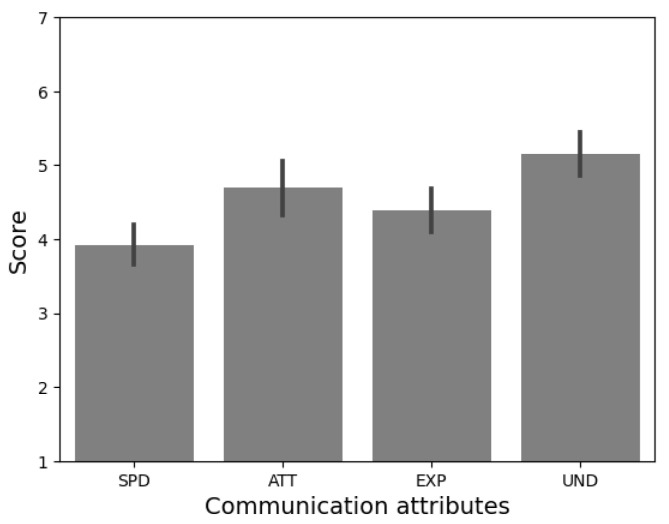
Responses to the communication attributes. Participants reported finding Qhali’s gestures as easy to understand.

Furthermore, as shown in Figure 18, participants consistently reported finding Qhali’s gestures easy to understand (*M* = 5.15, *SD* = 1.06). The interaction with Qhali was also perceived as attractive (*M* = 4.69, *SD* = 1.31) and rich in expressiveness (*M* = 4.38, *SD* = 1.04). However, as indicated in the preliminary validation, speed emerged as the least favorably rated attribute (*M* = 3.92, *SD* = 0.95).

Recent studies highlight the important role of movement speed in influencing the perception of dynamic gestures [55]. Current research underscores a human preference for slower robotic movements, associating this attribute with increased comfort and a sense of safety [55,56,57]. In light of these findings, our goal is to preserve the existing motion speed of Qhali, aligning with this identified preference to ensure user comfort and optimize gesture recognition. Furthermore, other studies, such as [58], have considered the recognition of human activity in order to determine the actions of a robot based on the predicted actions, using machine learning algorithms. This aspect has not been evaluated as part of this study but is a relevant topic for improving interaction with patients.

**Figure 19:**
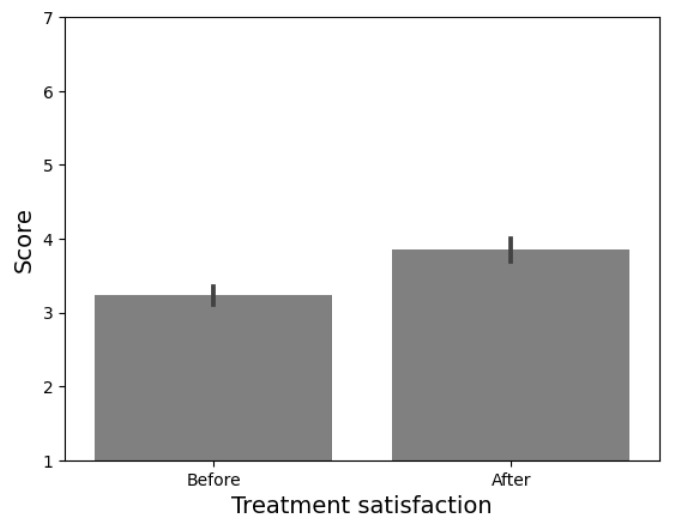
Differences in the treatment-perceived efficacy before and after the psychological intervention.

The perceived efficacy of treatment was calculated from the results of the CRES-4 scale (see Figure 19). From a range from 0 to 300, participants considered the treatment received as effective (*M* = 228.46, *SD* = 13.28). Nonetheless, the instrument provides insight into the emotional change before and after the application of the psychological intervention. To explore the possible differences in the satisfaction level, a within-subjects *t*-test was performed.

We found a significant difference in the emotional state before (*M* = 3.23, *SD* = 0.43) and after (*M* = 3.84, *SD* = 0.55) receiving psychological intervention *t*(12) = −4.338, *p* < 0.00, *d* = 1.2). These results suggest that the intervention delivered by Qhali may have positively influenced the emotional state of the participants.

## 6. Conclusions and Future Work

In this article, we present Qhali as an expressive humanoid robot designed to provide comprehensive assistance in the field of mental health. First, the industrial design of the humanoid robot is presented, describing the research, ideation, design, manufacturing, and implementation phases. Then, the components of the robot’s mechatronic system, including sensing, actuation, control, power, and software interface, are described. When the robot was fully operational, a behavioral experiment was conducted to validate Qhali’s capability of delivering telepsychological interventions.

Overall, Qhali stood out for its sympathy and engaging interaction with users, thanks to its anthropomorphic design, which was evident in both its physical attributes and in the design of its gestures and facial expressions. Furthermore, the incorporation of a playful script, characterized by its informative, rhetorical, and entertaining elements, contributes to the sense of pleasantness during the interaction. Moreover, it is worth noting that participants recognize the suitability of Qhali’s speed, a crucial aspect in HRI, as it significantly enhances user comfort and fosters a sense of perceived security.

All these factors combined yield valuable insights into the overall perceived effectiveness of the robot as a tool for delivering telepsychological interventions. This study reveals that users reported positive outcomes in their perception of the psychological intervention delivered by Qhali, highlighting an improvement in the user’s emotional state, noticeable both before and after their interaction with the robot.

The present pre-experimental study represents the initial phase of validating Qhali’s ability to deliver psychological interventions. In a small-sized group, we assessed important attributes for HRI, such as likeability and perceived intelligence; however, not all scores were uniformly high. Consequently, based on the insights gained from this study, our next step will be to conduct a larger validation study with a control group and a larger participant pool.

In the field of mental health, the applications of the Qhali robot are exponential. As seen above, robots are highly efficient in delivering telepsychological interventions, minimizing the sense of threat, and ensuring engagement with users. Qhali, with its tactile screen and intuitive interface, can conduct initial screenings to assess users’ mental states. Moreover, its design and interactive features allow for the dissemination of mental health promotion messages. Future work will aim to elevate Qhali as a multifaceted tool that addresses both physical and mental health. To achieve this objective, there are plans to integrate vital signal sensors, including temperature and blood pressure into Qhali’s hands.

These functionalities are not confined to a single setting; they can be adapted across various environments. This includes hospitals, nursing homes, schools, universities, and any workplace where employees encounter psychological stressors in their duties. Furthermore, they can be invaluable in remote areas with limited access to specialized mental health professionals, such as mining camps.

## Figures and Tables

**Table 1:** Movement ranges of robot articulations.

Name of Movement	Articulation	Movement Range
Neck Rotation	Head–Neck	− 90° to 90°
Neck Flexion	Head–Neck	−45° to 45°
Shoulder Flexion/Extensión	Shoulder	−60° to 120°
Shoulder Abduction	Shoulder	+0° to 120°
External/Internal Rotation	Forearm	−90° to 90°
Elbow Flexion	Elbow	−20° to 110°

## Data Availability

The data presented in this study are available upon request from the corresponding author.
